# A high-density genetic map of *Arachis duranensis*, a diploid ancestor of cultivated peanut

**DOI:** 10.1186/1471-2164-13-469

**Published:** 2012-09-11

**Authors:** Ervin D Nagy, Yufang Guo, Shunxue Tang, John E Bowers, Rebecca A Okashah, Christopher A Taylor, Dong Zhang, Sameer Khanal, Adam F Heesacker, Nelly Khalilian, Andrew D Farmer, Noelia Carrasquilla-Garcia, R Varma Penmetsa, Douglas Cook, H Thomas Stalker, Niels Nielsen, Peggy Ozias-Akins, Steven J Knapp

**Affiliations:** 1 Institute of Plant Breeding, Genetics and Genomics, University of Georgia, 111 Riverbend Rd, Athens, GA, 30605, USA; 2 National Center for Genome Resources, 2935 Rodeo Park Drive East, Santa Fe, NM, 87505, USA; 3 Department of Plant Pathology, University of California, Davis, CA, 95616, USA; 4 Department of Crop Science, North Carolina State University, Raleigh, NC, 27695, USA; 5 Department of Horticulture, University of Georgia, Tifton, GA, 31793, USA

## Abstract

**Background:**

Cultivated peanut (*Arachis hypogaea*) is an allotetraploid species whose ancestral genomes are most likely derived from the A-genome species, *A. duranensis*, and the B-genome species, *A. ipaensis*. The very recent (several millennia) evolutionary origin of *A. hypogaea* has imposed a bottleneck for allelic and phenotypic diversity within the cultigen. However, wild diploid relatives are a rich source of alleles that could be used for crop improvement and their simpler genomes can be more easily analyzed while providing insight into the structure of the allotetraploid peanut genome. The objective of this research was to establish a high-density genetic map of the diploid species *A. duranensis* based on *de novo* generated EST databases. *Arachis duranensis* was chosen for mapping because it is the A-genome progenitor of cultivated peanut and also in order to circumvent the confounding effects of gene duplication associated with allopolyploidy in *A. hypogaea*.

**Results:**

More than one million expressed sequence tag (EST) sequences generated from normalized cDNA libraries of *A. duranensis* were assembled into 81,116 unique transcripts. Mining this dataset, 1236 EST-SNP markers were developed between two *A. duranensis* accessions, PI 475887 and Grif 15036. An additional 300 SNP markers also were developed from genomic sequences representing conserved legume orthologs. Of the 1536 SNP markers, 1054 were placed on a genetic map. In addition, 598 EST-SSR markers identified in *A. hypogaea* assemblies were included in the map along with 37 disease resistance gene candidate (RGC) and 35 other previously published markers. In total, 1724 markers spanning 1081.3 cM over 10 linkage groups were mapped. Gene sequences that provided mapped markers were annotated using similarity searches in three different databases, and gene ontology descriptions were determined using the Medicago Gene Atlas and TAIR databases. Synteny analysis between *A. duranensis, Medicago* and *Glycine* revealed significant stretches of conserved gene clusters spread across the peanut genome. A higher level of colinearity was detected between *A. duranensis* and *Glycine* than with *Medicago*.

**Conclusions:**

The first high-density, gene-based linkage map for *A. duranensis* was generated that can serve as a reference map for both wild and cultivated *Arachis* species. The markers developed here are valuable resources for the peanut, and more broadly, to the legume research community. The A-genome map will have utility for fine mapping in other peanut species and has already had application for mapping a nematode resistance gene that was introgressed into *A*. *hypogaea* from *A*. *cardenasii*.

## Background

Cultivated peanut (*Arachis hypogaea* L.) is a major crop in most tropical and subtropical areas of the world and provides a significant source of oil and protein to large segments of the population in Asia, Africa and the Americas. In the U. S., peanut is a high-value cash crop of regional importance, with major production areas concentrated in the Southeast. Plant breeding efforts to pyramid genes for disease and insect resistances, quality, and yield is hampered by the polyploid genetics of the crop species, the multigenic nature of many traits (e.g., yield), and the difficulty of selecting for many traits in the field (e.g., soil borne diseases). Thus, secondary selection methods that are environmentally neutral would greatly facilitate crop improvement efforts. Molecular markers fit this criterion, but only recently have markers been developed that reveal sufficient polymorphisms in *A. hypogaea* and related species to have wide-spread application in peanut breeding. Preliminary steps for utilizing molecular markers for crop improvement are developing collections of polymorphic markers and utilizing them to construct dense and high-resolution genetic maps.

Constructing a high-quality genetic map depends largely upon finding one or more marker systems that can detect high levels of polymorphism between two individual parents. Unfortunately, low levels of molecular polymorphism were observed within tetraploid (2*n =* 4*x =* 40) *A. hypogaea* throughout the 1990s and early 2000s with the marker systems available at that time [1,2]. However, compared with the limited numbers of polymorphic markers detected for the tetraploid, the same marker systems can uncover high levels of molecular polymorphism within and between the diploid (2*n =* 2*x =* 20) peanut species. This polymorphism led researchers to create molecular maps for *Arachis.* The first molecular map in peanut was constructed between the diploids *A. stenosperma* Krapov. and W.C. Gregoryx and *A. cardenasii* Krapov. and W.C. Gregory by Halward et al. [3] who used Restriction Fragment Length Polymorphisms (RFLPs) to associate 117 markers into 11 linkage groups. Additional maps were subsequently published using Randomly Amplified Polymorphic DNA (RAPD) [4] and Simple Sequence Repeats (SSRs) [5,6]. Burow et al. [7] published the first tetraploid map in peanut based on 370 RFLP loci across 23 linkage groups by utilizing the complex interspecific cross, Florunner × 4x *A. batizocoi* Krapov. and W.C. Gregory (*A. cardenasii* × *A. diogoi* Hoehne)]. Another interspecific tetraploid linkage map of 298 loci and 21 linkage groups was derived from a backcross population between *A. hypogaea* and a synthetic amphidiploid [8]. Only recently have linkage maps been developed from crosses between *A. hypogaea* genotypes, most with less than 200 loci and with more than the expected 20 linkage groups [9-13]. An exception is the recently published map containing 1114 loci across 21 linkage groups that was constructed in part with highly polymorphic markers derived from sequences harboring miniature inverted repeat transposable elements [14]. Therefore, there is a continuing need to generate dense linkage maps for the cultivated tetraploid peanut that will not only cluster the markers into the expected 20 linkage groups to cover the haplotype chromosomes, but also to facilitate marker-trait association and eventually assist in its genetic improvement.

The domesticated peanut is thought to have arisen from a single hybridization event between two diploid wild species followed by whole genome duplication approximately 3,500 years ago [15]. This short evolutionary history, along with hybridization barriers between diploids and the tetraploid have resulted in a narrow genetic base for the cultivated tetraploid peanut. On the contrary, diploid *Arachis* species are genetically diverse, have simpler inheritance patterns, and most importantly, contain a rich source of agronomically important traits for peanut improvement. Due to these attributes, diploid *Arachis* species have been proposed as model systems to map the peanut genome. Because the genomes of progenitor diploid species [i.e., *A. duranensis* (A-genome donor) and *A. ipaensis* (B-genome donor)] are closely allied to the cultivated peanut [16], mapping the genome of one or both of these species should be useful for predicting the positions of loci in the cultivated peanut. This approach has been employed in wheat [17,18], alfalfa [19,20], oat [21], and other crop species.

One accession of *A*. *ipaensis* and 67 accessions of *A*. *duranensis* have been collected in South America. The largest concentration of *A*. *duranensis* is in southern Bolivia and northern Argentina, with a few populations being reported in Paraguay and one in central Brazil [22,23]. The species is morphologically diverse and the Bolivia and Argentina types can be separated cytogenetically and morphologically [24]. Due to the availability of diverse accessions to produce intraspecific crosses in the greenhouse, a dense linkage map in the diploid species *A. duranensis* was produced using large numbers of molecular markers derived from transcribed sequences.

## Results and discussion

### Species relationships

**Figure 1:**
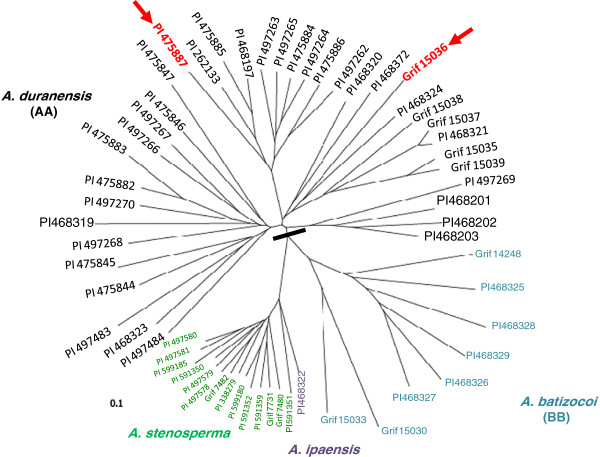
**Genetic relationships among A- and B-genome *Arachis *species.** Clustering of A- (*A. duranensis* and *A. stenosperma*) and B- (*A. ipaensis* and *A. batizocoi*) genome species according to analysis of data from SSR markers. The two parents used for mapping are indicated by arrows.

A preliminary study of SSR marker variation among 37 *A. duranensis* accessions using 556 markers indicated that the species is highly polymorphic at the molecular level and individual accessions could be separated based on a cluster analysis (Figure 1). Interestingly, we found that *A. ipaensis*, the proposed B-genome (BB) progenitor species, clustered with the A-genome (AA) species *A. stenosperma* and not with the B-genome species *A. batizocoi.* Recent molecular cytogenetic analysis of A- and non-A- (i.e., B-) genome species suggests that karyotype diversity among non-A-genome species is extensive enough to support separation into additional genome classes where *A. ipaensis* remains in B *sensu stricto* while *A. batizocoi* is placed into a separate group [25]. Therefore, *A. batizocoi* is less typical of B-genome species.

The number of polymorphic SSR markers between paired *A. duranensis* accessions ranged from 160 to 375 out of 556, which is 29 to 67% of the total number of SSR markers screened. This is a significant amount of variation, which indicates the high genetic diversity within the species. Based on cluster analysis, success of crosses, and fertility of F_1_s, accessions PI 475887 and Grif 15036 were selected for subsequent mapping studies using 94 F_2_ progenies. Screening of the parental accessions with 2,138 SSR markers derived from *A. hypogaea* EST sequences resulted in 1,768 (82.7%) that were scorable (detected by ABI3730XL genotyping systems) and 896 (41.9%) that were polymorphic (Guo Y et al: Comparative mapping in intraspecific populations uncovers a high degree of macrosynteny between A- and B-genome diploid species of peanut, Submitted). The same markers were used to create a map between two *A. batizocoi* accessions and to determine syntenic relationships between the A and B genome species (Guo Y et al: Comparative mapping in intraspecific populations uncovers a high degree of macrosynteny between A-and B-genome diploid species of peanut, submitted).

### *Arachis duranensis* genetic map

**Figure 2:**
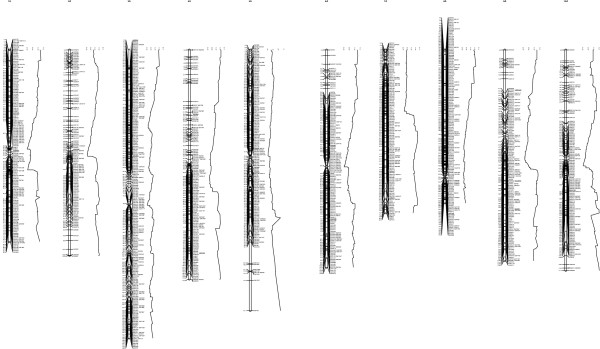
**High-density linkage map of *Arachis duranensis *including 1,724 markers.** SNP and SSR markers are prefixed by ‘SNP’ and ‘GM’, respectively, resistance gene candidate markers are prefixed by ‘RGC’ and ‘GS’. Twenty-four previously published markers (underlined) were selected from an interspecific map between *A. duranensis* and *A. stenosperma*[36] to establish synteny between the current and former linkage groups. The original linkage group assignments are given in the marker names separated by the pound (#) sign. Loci with significant segregation distortion (p = 0.05) are labeled with an asterisk. Graphs to the right of the linkage groups represent recombination frequencies. Each data point represents genetic distances between adjacent markers averaged for a window of 20 markers.

The total number of published SSR markers has now risen beyond the 2,847 cataloged in a related paper by Guo et al. (Guo Y et al: Comparative mapping in intraspecific populations uncovers a high degree of macrosynteny between A-and B-genome diploid species of peanut, submitted) to around 6,000 [26]. Those most recently reported include: 14 by Gimenes et al. [27]; 51 by Mace et al. [28]; 188 by Proite et al. [29]; 104 by Cuc et al. [30]; 138 by Yuan et al. [31]; 33 by Song et al. [32]; 123 by Wang et al. [33]; 290 by Liang et al. [34]; and 1,571 by Koilkonda et al. [35]. Five hundred and ninety-eight of these markers are included in the *A. duranensis* map (Figure 2). Of the 34 genomic SSR markers mapped in the current study (Table 1), 24 were mapped previously in an interspecific population between *A. duranensis* and *A. stenosperma*[6,36]. These markers served to anchor and align the current and previously published peanut maps (Figure 2). Linkage group assignments of all markers were consistent between the current map and that of Bertioli et al. [36] except for the marker GM117 (AC3C02 on map in reference 36 derived from GenBank accession DQ099133) that was localized on chromosome 2A (the ‘A’ following a chromosome number is presented in this study to represent chromosomes in the A genome of peanut) in their interspecific map, while mapping to chromosome 10A in the *A. duranensis* intraspecific map. Although detailed information for parental alleles in the study by Bertioli et al. [36] was not presented, GM117 amplified only one locus from each parent in both their population and ours. It is, therefore, unlikely that the marker location discrepancy was due to mapping of multiple loci and perhaps could reflect a small chromosomal rearrangement. Chromosomal rearrangements are not unexpected based on previous cytological observations in the genus [24,37].

**Table 1:** **Previously published genomic SSR markers mapped in*Arachis duranensis***

**Universal Name**	**Original Name**	**Forward (5′-3′)**	**Reverse (5′-3′)**	**Reference**
GM7	Ah1TC1D02	GATCCAAAATCTCGCCTTGA	GCTGCTCTGCACAACAAGAA	Moretzsohn et al. 2005
GM10	Ah1TC1E05	GAAGGATAAGCAATCGTCCA	GGATGGGATTGAACATTTGG	Moretzsohn et al. 2005
GM13	Ah1TC1H04	CATTACTTCCTAGGTTTGTTTTCCA	ATGGCGTGACAACGGAAC	Moretzsohn et al. 2005
GM16	Ah1TC2B01	TTGCAGAAAAGGCAGAGACA	GAAAGAAGCTAAGAAGGACCCATA	Moretzsohn et al. 2005
GM19	Ah1TC2C07	CACCACACTCCCAAGGTTTT	TCAAGAACGGCTCCAGAGTT	Moretzsohn et al. 2005
GM22	Ah1TC2D06	AGGGGGAGTCAAAGGAAAGA	TCACGATCCCTTCTCCTTCA	Moretzsohn et al. 2005
GM24	Ah1TC2E05	GAATTTATAAGGCGTGGCGA	CCATCCCTTCTTCCTTCACA	Moretzsohn et al. 2005
GM28	Ah1TC3A12	GCCCATATCAAGCTCCAAAA	TAGCCAGCGAAGGACTCAAT	Moretzsohn et al. 2005
GM32	Ah1TC3E02	TGAAAGATAGGTTTCGGTGGA	CAAACCGAAGGAGGAACTTG	Moretzsohn et al. 2005
GM38	Ah1TC3H02	CTCTCCGCCATCCATGTAAT	ATGGTGAGCTCGACGCTAGT	Moretzsohn et al. 2005
GM58	Ah1TC4G06	ATTTCACATTCCCTAGCCCC	CATCGACTGACTTGAAAAATGG	Moretzsohn et al. 2005
GM59	Ah1TC4G10	TTCGGTCATGTTTGTCCAGA	CTCGAGTGCTCACCCTTCAT	Moretzsohn et al. 2005
GM66	Ah1TC5D06	GAAATTTTAGTTTTCAGCACAGCA	TTTTCCCCTCTTAAATTTTCTCG	Moretzsohn et al. 2005
GM68	Ah1TC6E01	CTCCCTCGCTTCCTCTTTCT	ACGCATTAACCACACACCAA	Moretzsohn et al. 2005
GM69	Ah1TC6G09	GGAGGTTGCATGCATCATAGT	TCATTGAACGTATTTGAAAGCTC	Moretzsohn et al. 2005
GM71	Ah2TC7A02	CGAAAACGACACTATGAAACTGC	CCTTGGCTTACACGACTTCCT	Moretzsohn et al. 2005
GM74	Ah2TC7E04	GAAGGACCCCATCTATTCAAA	TCCGATTTCTCTCTCTCTCTCTC	Moretzsohn et al. 2005
GM76	Ah2TC7G10	AATGGGGTTCACAAGAGAGAGA	CCAGCCATGCACTCATAGAATA	Moretzsohn et al. 2005
GM83	Ah2TC9C06	CAAATGGCAGAGTGCGTCTA	CCCTCCTGACTGGGTCCT	Moretzsohn et al. 2005
GM92	Ah2TC11A04	ACTCTGCATGGATGGCTACAG	CATGTTCGGTTTCAAGTCTCAA	Moretzsohn et al. 2005
GM96	Ah2TC11C06	TCCAACAAACCCTCTCTCTCT	GAACAAGGAAGCGAAAAGAA	Moretzsohn et al. 2005
GM117	Ah2AC3C02	TCTAACGCACACAAATCGAA	CTTGTACCTGCGCCATTCT	Moretzsohn et al. 2005
GM126	AS1RI1F06	TGTCTCTCTTCCTTTCCTTGCT	CCTTTTGCTTCTTTGCTTCC	Moretzsohn et al. 2005
GM162	AS1RN9C02	CGTTACACTGAGCCAGCAACTC	ACGGCGGCGATAGTTTCA	Moretzsohn et al. 2005
GM170	AS1RN11E05	CTCGGTCCAGAAAACACAGG	GTAGAGGCGAAGAAGGCAGAG	Moretzsohn et al. 2005
GM218	gi-30419832	GCCACTTTATTCTAAGCACTCC	AAGAGACCACACGCTCACA	Moretzsohn et al. 2005
GM226	gi-30419936	TCACAGATCCATAGACTTTAACATAGC	CCGGTGTGGATTCATAGTAGAG	Moretzsohn et al. 2005
GM255	pPGPSeq4H6	CCAACATTGCAGAAGCAAGA	CAAAGAGAGGCACACCACAA	Moretzsohn et al. 2005
GM286	Ah-193	CTTGCTGAAGGCAACTCCTACG	TCGGTTTGTCTCTTTGGTCACTC	Moretzsohn et al. 2004
GM324	Ah-649	GGAAATGCCAAATCCATCCTTC	GTTGTTCGGTGTGAAAACGGTC	Moretzsohn et al. 2004
GM328	Ah-671	AGAAAGAGCACGGGACATTACC	ATGAATGAGTGGTCATACGCGA	Moretzsohn et al. 2004
GM565	pPGSseq17E3	TTTCCTTTCAACCCTTCGTG	AATGAGACCAGCCAAAATGC	Ferguson et al. 2004
GM664	GM664	CTTCACCTCCAAAATCAACCA	ACCGCTGACATTTGATTGTTC	Guo et al. 2011
GM671	GM671	TGGATGCTGTAAGGAATGGAC	TTATCGAGCTTGCCTCAGAAA	Guo et al. 2011

Markers were renamed in order to follow a unified marker nomenclature. The complete list of renamed markers can be found in Guo et al. (Guo Y et al: Comparative mapping in intraspecific populations uncovers a high degree of macrosynteny between A-and B-genome diploid species of peanut, Submitted).

EST libraries of *A. duranensis* were developed to produce Single Nucleotide Polymorphism (SNP) markers for mapping (Table 2). Of the 1,536 SNP markers developed (Additional file 1), 1,054 were included in the *A. duranensis* map (Figure 2). The remaining 482 SNP markers were either of low quality (GC quality score <0.25) or they showed segregation patterns (extremely distorted) that could not be mapped. Of the 1,054 mapped SNP markers, 815 were derived from the cDNA sequencing project while the other 239 were genomic legume orthologs.

**Table 2:** cDNA sequence reads generated for single nucleotide polymorphism (SNP) discovery inArachis duranensis*

**Accession**	**Sequencing Platform**	**Tissue type**		**Total**
		**Developing seed**	**Root**	
PI 475887	Sanger	22,356	21,487	43,843
PI 475887	454	212,938	266,575	479,513
Grif 15036	454	296,242	235,245	531,487
**Total**		531,536	523,307	1,054,843

* Assembly is deposited at NCBI as Accession: PRJNA50587.

The *A. duranensis* map produced in this study contained 1,724 markers combined into 10 linkage groups with a total genetic distance of 1081.3 cM. MSTMap, a software program that accommodates large numbers of markers and utilizes a “minimum spanning tree” algorithm, was used to construct an initial genetic map using only the codominant markers. The 1,673 codominant markers were distributed into 810 co-segregating groups (bins). Although this program has been reported to be accurate for large-scale mapping projects [38], few independent studies are available establishing consistency between MSTMap and other commonly used mapping software [39]. To confirm the linkage group assignments, marker orders, and genetic distances determined by alternative software, both codominant and dominant markers were mapped with Joinmap 3.0. Marker orders and genetic distances were highly consistent between MSTMap and Joinmap 3.0 (Additional file 2).

Significant segregation distortion (p = 0.05) was observed for 513 (29.8%) markers (Figure 2, Additional file 3). Chromosomes 4A and 9A carried particularly long segments of distorted segregation suggesting large-scale chromosomal selection in these regions. Guo et al. (Guo Y et al: Comparative mapping in intraspecific populations uncovers a high degree of macrosynteny between A-and B-genome diploid species of peanut, Submitted) found that a single linkage group (4/9B) in *A. batizocoi* was syntenic with chromosomes 4A and 9A of *A. duranensis* implicating inversion and reciprocal translocation events as the underlying chromosomal rearrangements in this B-genome species. Recombination frequencies were generally low in the central, presumably centromeric chromosomal regions of *A. duranensis* and increased toward the telomeres, a pattern typical of many plant species [40,41]. More even distribution was observed along chromosome 3A and only slightly suppressed recombination was observed around the presumable location of the centromere (Figure 2).

Across the *A. duranensis* linkage map, each linkage group spanned on average 108.1 cM (77.3-145.6 cM) and included 172.4 markers (119–266) (Table 3). This is considerably denser than the previously published AA, BB, and AABB maps consisting of only a few hundred markers. For example, the *A. ipaensis × A. magna* B-genome map published by Moretzsohn et al. [5] had 149 SSR markers grouped into 10 linkages, whereas the B-genome SSR-based map in our related paper consists of 449 loci in 16 linkage groups (Guo Y et al: Comparative mapping in intraspecific populations uncovers a high degree of macrosynteny between A-and B-genome diploid species of peanut, Submitted). The A-genome map produced using the interspecific hybrid *A. duranensis* × *A. stenosperma* had 339 SSRs that were mapped into 11 linkage groups [6,42]. For *A. hypogaea,* there are now several maps with the most dense consensus map containing 324 loci on 21 linkage groups [11].

**Table 3:** **Genetic distances and distribution of markers on the ten linkage groups of *A. duranensis***

**Linkage group**	**Genetic distance (cM)**	**Markers**
1A	96.8	186
2A	103.9	119
3A	145.6	266
4A	115.8	149
5A	131.7	178
6A	109.8	181
7A	82.3	141
8A	77.3	180
9A	106.5	171
10A	111.4	153
**Total**	1081.3	1724

The map produced in the current study is the first high-density map available in peanut, and because it was generated from a progenitor species of *A. hypogaea*, we anticipate that it will have significant applications for analyzing the cultivated genome. For example, the data generated in this map was used by Nagy et al. [43] to more precisely map the *Rma* gene for nematode resistance that originated from an introgression line between *A. hypogaea* and *A. cardenasii*. The A-genome SNP array also has been useful at the tetraploid level for genotyping a recombinant inbred line population derived from a cross between cultivated peanut and a synthetic *A. ipaensis* x *A. duranensis* tetraploid (Ozias-Akins, unpublished).

### Gene annotation and comparative mapping

Homology search of the 1,724 mapped loci resulted in significant hits for 1,463 loci in at least one of the three databases: Medicago, Uniprot and GenBank NR database, and 580 of the mapped loci gave significant similarities in either of the two gene ontology databases: Medicago Gene Atlas and TAIR (Additional file 4). Altogether 1,366 gene ontology terms were assigned to the 580 genes. These were distributed among the three major gene ontology categories as follows: 521 molecular functions, 534 biological processes, and 311 cellular components (Additional file 4).

**Figure 3:**
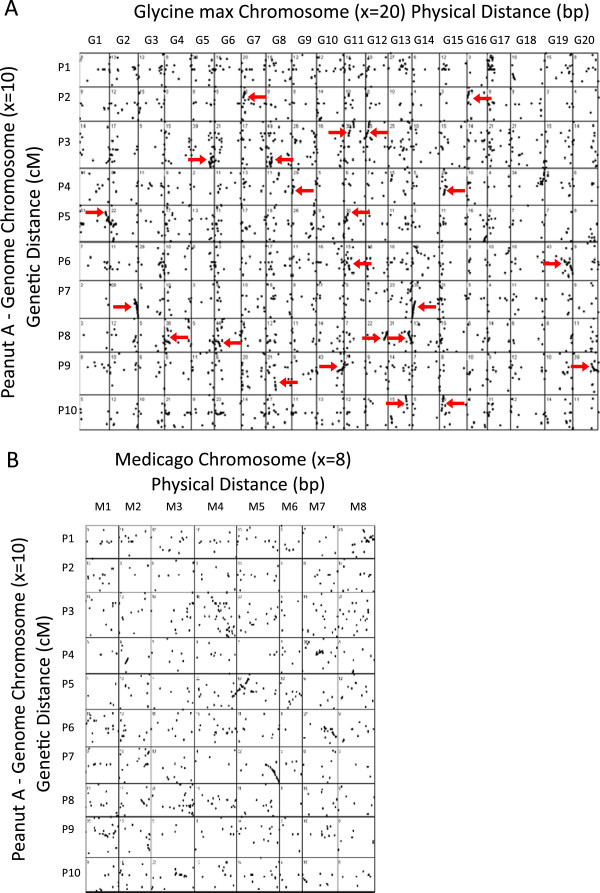
**Synteny between diploid A-genome peanut (*A. duranensis*, 2*n =*20) and *Glycine max *(2*n*= 40).** Arrows indicate clusters of genes in common between the two genomes. For plotting the data on the Y axis, the peanut genome for each chromosome is proportional in size to the total map size in centimorgans. For the X axis, the unit of measure is scaled to bp within the chromosomal assemblies of the respective genomes. The plot was obtained with a visual basic program that plotted the x‐y coordinates of each hit. The total number of matches for each pair wise comparison is listed at the upper left corner.

The sequences used to create the *A. duranensis* map also were compared to the genomes of two legumes where 995 loci on the *A. duranensis* map could be mapped to *M. truncatula*, and 2,711 matches could be found in *G. max* (with potentially two hits per mapped locus). While a majority of the dots in the synteny plots appear to be random (Figure 3), there are definite clusters of markers, and striking examples of colinearity (red arrows), especially for the comparisons to *Glycine.* Presumably there has been extensive single gene movement since the last common ancestors in one or both species, but many genes remain in the ancestral locations and can be detected. Overall, the synteny patterns for *G. max* showed the recent whole genome duplication within *Glycine*, with each location in peanut showing corresponding synteny at two locations in *Glycine*. Colinearity between *Medicago* and *Arachis* is much less conserved than between *Arachis* and *Glycine*. This could be due to extensive inversions in either genome, or more likely, due to preliminary ordering of sequences within the *Medicago* unfinished genome assembly. In general, the patterns showed strong synteny on the chromosomal ends in both genetic and physical distance, while the central regions of chromosomes tended to show less synteny. Presumably this could be attributed to pericentromeric heterochromatin which is known to define less recombinogenic regions where genomic rearrangements are more likely to persist [44]. Chromosome arms tend to be maintained as syntenic between *Glycine* and *Arachis*, but there is evidence that chromosome arms have been translocated in some cases so that synteny exists at the chromosome arm level, but less so at the whole chromosome level.

## Conclusions

This investigation provided a large number of de novo EST sequences that were deposited into GenBank. The markers developed here are valuable resources for peanut and, more broadly to the legume research community. This research presents the first high-density molecular map in peanut with 1,724 markers grouped into the 10 expected linkage groups for an A-genome species. Because the map was produced with the progenitor species *A*. *duranensis* which contributed the A genome of *A*. *hypogaea*, it will serve as the reference map for both wild and cultivated species. Lastly, synteny was found between *Arachis* and the *Glycine* and *Medicago* genomes, which indicates that markers developed for other legume species may be of value for crop improvement in peanut. The A-genome map will have utility for fine mapping in other peanut species and has already had application to mapping a nematode resistance gene that was introgressed to *A*. *hypogaea* from *A*. *cardenasii*.

## Methods

### Plant materials

Thirty-seven accessions of *A. duranensis*, 14 accessions of *A. stenosperma* (A genome), one accession of *A. ipaensis*, and eight accessions of *A. batizocoi* (B genome) were obtained from the USDA or NCSU germplasm collections. Plants were then grown in greenhouses at the University of Georgia at Athens. The accessions evaluated are shown in Figure 1. Hybrids were made between three pairs of *A. duranensis* accessions, including PI 468200 × PI 468198, PI 468319 × PI 475885, and PI 475887 × Grif 15036. The hybrid combination PI 475887 × Grif 15036 was one of the most polymorphic as revealed by using a panel of SSR markers as described below and thus was selected for subsequent mapping. PI 475887 was originally collected by Krapovickas, Schinini, and Simpson near Salta, Argentina during 1980, and Grif 15036 was originally collected by Williams, Simpson, and Vargas near Boqueron, Paraguay during 2002 [22]. Crosses were made by manually emasculating flowers of the female parent PI 475887 during the late afternoon and pollinating stigmas between 8 and 10 am the following morning with pollen from the male parent Grif 15036. An F_2_ population was developed by self-pollinating multiple F_1_ individuals. The intraspecific F_2_ population (*n* = 94) from a hybrid between two *A. duranensis* accessions was then used for mapping studies.

### Molecular diversity between and within A- and B-genome diploid species

DNA was isolated from leaf samples of *A. duranensis, A. ipaensis, A. stenosperma,* and *A. batizocoi* accessions using a modified CTAB method [45,46]. The 60 DNA samples were amplified using 709 different SSR primer pairs (GM1-GM709) that had been generated from sequences reported in the literature [6,29,47-53] and screened for polymorphisms. SSR markers were genotyped on an ABI3730XL Capillary DNA Sequencer (Applied Biosystems, Foster City, CA) as described in a related paper by Guo et al. (Guo Y et al: Comparative mapping in intraspecific populations uncovers a high degree of macrosynteny between A-and B-genome diploid species of peanut, Submitted) using forward primers labelled with FAM, HEX, or TAMRA fluorophores. Microsat [54] was used for construction of a distance matrix based on the proportion of shared bands (D = 1 - ps) from 556 primer pairs amplifying polymorphic fragments. The matrix was imported into Phylip v3.67 [55] for the construction of the neighbor-joining tree.

### Marker development

#### Simple sequence repeat (SSR) markers

A total of 101,132 unigenes (37,916 contigs (GenBank Acc. No. EZ720985-EZ758900) and 63,216 singletons) from tetraploid peanut ESTs (GenBank Acc. No. CD037499-CD038843, ES702769-ES768453, GO256999-GO269325, GO322902- GO343529 and short-read Sequence Read Archive accessions SRX020012, SRX019979, SRX019972, SRX019971) representing ca. 37 Mb of the *A. hypogaea* genome were mined for 2,138 EST-SSR markers (GM710-GM2847) (Guo Y et al: Comparative mapping in intraspecific populations uncovers a high degree of macrosynteny between A-and B-genome diploid species of peanut, Submitted). Unigenes in the transcript assembly were screened for perfect repeat motifs using SSR-IT http://www.gramene.org/db/markers/ssrtool) and for imperfect motifs using FastPCR (http://primerdigital.com/fastpcr.html). The repeat count (n) threshold for each motif type was set for n ≥ 5. SSR markers were genotyped on an ABI3730XL Capillary DNA Sequencer (Applied Biosystems, Foster City, CA) using forward primers labelled with FAM, HEX, or TAMRA fluorophores. PCR was performed in a 12 μL reaction mixture containing 1.0 × PCR buffer, 2.5 mM Mg^++^, 0.2 mM each of dNTPs, 5.0 pmol of each primer, 0.5 unit of *Taq* polymerase, and 10 ng of genomic DNA. Touchdown PCR was used to reduce spurious amplification. The SSR markers were screened for length polymorphisms using GeneMapper 3.0 software (Applied Biosystems, Foster City, CA). Of the 2,138 EST-SSR primer pairs tested, markers derived from 598 could be mapped. A set of 34 SSR markers from genomic sequences of *Arachis* previously screened for polymorphism between parents of the *A. duranensis* mapping population (Guo Y et al: Comparative mapping in intraspecific populations uncovers a high degree of macrosynteny between A-and B-genome diploid species of peanut, Submitted) were also mapped (Table 1).

#### Single-stranded conformational polymorphism (SSCP) markers

SSCP markers were developed from genomic DNA templates for previously described NBS sequences isolated by targeting conserved sequence motifs in NBS-LRR encoding genes [56,57] and from *Arachis* unigenes showing similarity to R-gene homologs identified by mining a peanut transcript assembly [43]. SSCP fragments were amplified using touch-down PCR and detected by silver-staining as previously described [58-60]. A total of 380 SSCP markers were evaluated for polymorphism between the parents PI 475887 and Grif 15036. The resistance gene analog markers are prefixed by either ‘GS’ or ‘RGC’ in the map. cDNA sequences for unigenes targeted for SSCP marker development in the present study were deposited in GenBank (Acc. No. GF100476-GF100638). One additional marker, the SCAR marker S197 linked to a root-knot nematode resistance gene in *Arachis hypogaea*[43,61] was also mapped.

#### Development of single nucleotide polymorphism (SNP) markers

Total RNA was isolated from roots of young seedlings (up to four trifoliate) and from developing seeds (up to developmental stage R6) of the two parental genotypes, PI 475887 and Grif 15036 (alias DUR25 and DUR2, respectively). cDNA libraries were developed using the Mint cDNA synthesis kit (Evrogen) and normalized using the Trimmer cDNA normalization kit (Evrogen). cDNA sequences were generated by Sanger and 454 GS-FLX sequencing methods and assembled using the tool Mira [62]. Altogether, more than one million cDNA sequence reads were generated from *A. duranensis* PI 475887 and Grif 15036. These were assembled into 81,116 unique transcripts (unigenes) (GenBank Accn. No. HP000001-HP081116). Assemblies were searched for single nucleotide polymorphisms (SNPs) that fulfilled the following two criteria: (a) the SNP position is covered at least by two reads from each genotype, and (b) at least 80% of the reads call the SNP in the particular genotype. Using these criteria, we identified 8,478 SNPs in 3,922 unigenes. To facilitate the selection of candidate SNPs for designing and building Illumina GoldenGate SNP genotyping arrays, putative intron positions were predicted by aligning *Arachis* contigs with *Arabidopsis* and *Medicago* genomic DNA sequences identified by BLAST analyses. SNPs within 60 bp of a putative intron were eliminated, thereby reducing the collection of candidate SNPs to 6,789 in 3,264 unigenes from which 1,236 high-quality SNPs, each representing separate unigenes, were selected for genotyping. SNPs were also detected by allele re-sequencing in a subset of 768 conserved legume orthologs identified by coauthors (R.V. Penmetsa, N. Carrasquilla-Garcia, A. D. Farmer and D.R. Cook), and 300 of these SNPs were added to the GoldenGate array. SNP genotyping on the GoldenGate array was conducted at the Emory Biomarker Service Center, Emory University. The BeadStudio (Illumina) genotyping module was used for calling genotypes. Markers with GC quality scores lower than 0.25 were excluded from subsequent analysis.

### Map construction

The program, MSTMap [39] was used to build a core genetic map including all codominant markers using the cut-off p-value of 10^-12^ for clustering markers into linkage groups. The recombinant inbred line2 (RIL2) algorithm and Kosambi function were used to calculate genetic distances. The program Joinmap 3.0 [63] was used to localize the dominant markers and to confirm the marker order, a range of LOD scores of 5–16 was used to create groups. The Kosambi mapping function was used for map length estimations. Markers were tested for segregation distortion by the chi-square test. Graphic presentation of the map was drawn using Mapchart 2.0 software [64].

### Gene annotation

The cDNA sequences included in the genetic map have been used to search for homologous genes in the Medicago (http://www.medicago.org), Uniprot (http://www.uniprot.org) and GenBank NR (http://www.ncbi.nlm.nih.gov/genbank) databases using various blast algorithms. Gene ontology annotations were also added by searching Medicago Gene Atlas (http://mtgea.noble.org) and The Arabidopsis Information Resource (TAIR, http://www.arabidopsis.org) databases. A significance threshold of E =1e-5 was applied in all inquiries.

### Synteny between *Arachis*, *Medicago*, and *Glycine*

The EST sequences used for marker-development were compared to the whole genome sequences of *Glycine max* and *Medicago truncatula* to establish synteny. Sequences for the genomes *G. max* V5 and *M. truncatula* MT3.0 were obtained through http://www.phytozome.net. The sequences associated with each locus on the *A. duranensis* peanut map (Additional file 1 and Additional file 5) were searched against the respective whole genome sequences using blastn and E < =1e-6. For comparison to *Medicago*, only the best match was retained because diploid peanut and *M. truncatula* are at the same relative ploidy level. However for *Glycine*, the two best matches for each peanut sequence were retained because of the recent polyploidy within soybean and the high level of retention of duplicated genes in the species. Blast hits to scaffolds or Bacterial Artificial Chromosomes (BACs) not anchored to the chromosomal assembly in the target genomes were discarded. Plotting the data and processing of blast results were performed with Visual Basic programs written for this study.

## Competing interests

The authors declare that they have no competing interests.

## Authors’ contributions

EDN developed the cDNA libraries, organized sequencing and SNP genotyping, and constructed the initial genetic maps. YG conducted parental genotyping and constructed final genetic maps. EDN and ST developed the mapping population. EDN and RAO genotyped the SSR, RGC, and RS markers. JEB performed the synteny analysis. ST, YG, SK, AFH, NK developed the SSR markers. SK performed the genetic diversity study. CAT and DZ assembled the ESTs and nominated SNPs for the genic sequences. ADF, NCG, RVP and DC nominated the conserved legume ortholog SNPs. EDN and JEB participated in manuscript writing. DC, HTS and NN contributed to project design. POA was Co-PI of the project and finalized the manuscript. SJK was the PI of the project, designed experiments, coordinated the study, and participated in manuscript writing. All authors read and approved the final manuscript.

## Supplementary Material

Additional file 1 **SNP markers on Illumina GoldenGate array.** Marker ID along with sequence information for OPAs and target ESTs are provided. Click hereb for file

Additional file 2 **Comparative genetic mapping of*Arachis duranensis*using two different software programs on the same dataset.** Genetic maps were constructed by MSTMap (left) using 1673 co-dominant markers and Joinmap 3.0 (right) using 1724 markers. Linkage group assignments, marker orders and genetic distances were highly consistent, except for the order among a few closely linked loci. Marker positions determined by Joinmap 3.0 are provided in Additional file 6. Click here for file

Additional file 3 **Mapped markers with segregation distortion (p = 0.05) and their position on the map.** Column A lists the marker positions on each chromosome, column B indicates marker name, column C is the chromosome number, columns D to H list the number of individuals in each genotype class, and columns J and K provide χ^2^ and P values, respectively. Click here for file

Additional file 4 **Annotated loci mapped in*Arachis duranensis.*** Columns B to J include homologs identified in the Medicago (B-D), GenBank-NR (E-G) and Uniprot-Sprot (H- J) databases. Columns L to S include gene ontology (GO) terms identified in the three major GO categories: molecular function (N, O), biological process (P,Q) and cellular component (R,S). Click here for file

Additional file 5 **Sequences associated with SSR and SSCP markers for synteny analysis.** Column A is the marker name and column B is the Genebank ID or sequence used as query for synteny analysis. Click here for file

Additional file 6 Marker positions for the linkage map. Click here for file

## References

[B1] Stalker HT Mozingo LG Molecular markers of Arachis and marker-assisted selection Peanut Sci 2001 28 117 123 10.3146/i0095-3679-28-2-13

[B2] Paterson AH Stalker HT Gallo-Meagher M Burow MD Dwivedi SL Crouch JH Mace ES Wilson RF, Stalker HT, Brummer CE Genomics and genetic enhancement of peanut Genomics for Legume Crops 2004 Amer Oil Chem Soc, Champaign, IL 97 109

[B3] Halward T Stalker HT Kochert G Development of an RFLP linkage map in diploid peanut species Theor Appl Genet 1993 87 379 384 10.1007/BF01184927 24190266

[B4] Garcia GM Stalker HT Schroeder E Lyerly JH Kochert G A RAPD-based linkage map of peanut based on a backcross population between the two diploid species of Arachis stenosperma and A. cardenasii Peanut Sci 2005 32 1 8 10.3146/0095-3679(2005)32[1:ARLMOP]2.0.CO;2

[B5] Moretzsohn MC Barbosa AVG ves-Freitas DMT Teixeira C Leal-Bertioli SCM Guimaraes PM Pereira RW Lopes CR Cavallari MM Valls JFM A linkage map for the B-genome of Arachis (Fabaceae) and its synteny to the A-genome BMC Plant Biol 2009 9 40 10.1186/1471-2229-9-40 19351409 PMC2674605

[B6] Moretzsohn MC Leoi L Proite K Guimaraes PM Leal-Bertioli SCM Gimenes MA Martins WS Valls JFM Grattapaglia D Bertioli DJ A microsatellite-based, gene-rich linkage map for the AA genome of Arachis (Fabaceae) Theor Appl Genet 2005 111 1060 1071 10.1007/s00122-005-0028-x 16088397

[B7] Burow MD Simpson CE Starr JL Paterson AH Transmission genetics of chromatin from a synthetic amphidiploid to cultivated peanut (Arachis hypogaea L.): Broadening the gene pool of a monophyletic polyploid species Genetics 2001 159 823 837 11606556 10.1093/genetics/159.2.823 PMC1461827

[B8] Fonceka D Hodo-Abalo T Rivallan R Faye I Sall MN Ndoye O Favero AP Bertioli DJ Glaszmann JC Courtois B Genetic mapping of wild introgressions into cultivated peanut: a way toward enlarging the genetic basis of a recent allotetraploid BMC Plant Biol 2009 9 103 10.1186/1471-2229-9-103 19650911 PMC3091533

[B9] Varshney RK Bertioli DJ Moretzsohn MC Vadez V Krishnamurthy L Aruna R Nigam SN Moss BJ Seetha K Ravi K The first SSR-based genetic linkage map for cultivated groundnut (Arachis hypogaea L.) Theor Appl Genet 2009 118 729 739 10.1007/s00122-008-0933-x 19048225

[B10] Hong YB Chen XP Liang XQ Liu HY Zhou GY Li SX Wen SJ Holbrook CC Guo BZ A SSR-based composite genetic linkage map for the cultivated peanut (Arachis hypogaea L.) genome BMC Plant Biol 2010 10 17 10.1186/1471-2229-10-17 20105299 PMC2835713

[B11] Qin H Feng S Chen C Guo Y Knapp S Culbreath A He G Wang M Zhang X Holbrook CC Ozias-Akins P Guo B An integrated genetic linkage map of cultivated peanut (Arachis hypogaea L.) constructed from two RIL populations Theor Appl Genet 2011 124 653 664 22072100 10.1007/s00122-011-1737-y

[B12] Gautami B Pandey MK Vadez V Nigam SN Ratnakumar P Krishnamurthy L Radhakrishnan T Gowda MVC Narasu ML Hoisington DA Quantitative trait locus analysis and construction of consensus genetic map for drought tolerance traits based on three recombinant inbred line populations in cultivated groundnut (Arachis hypogaea L.) Mol Breed 2011 30 757 772 22924017 10.1007/s11032-011-9660-0 PMC3410028

[B13] Ravi K Vadez V Isobe S Mir RR Guo Y Nigam SN Gowda MVC Radhakrishnan T Bertioli DJ Knapp SJ Identification of several small main-effect QTLs and a large number of epistatic QTLs for drought tolerance related traits in groundnut (Arachis hypogaea L.) Theor Appl Genet 2011 122 1119 1132 10.1007/s00122-010-1517-0 21191568 PMC3057011

[B14] Shirasawa K Koilkonda P Aoki K Hirakawa H Tabata S Watanabe M Hasegawa M Kiyoshima H Suzuki S Kuwata C In silico polymorphism analysis for the development of simple sequence repeat and transposon markers and construction of linkage map in cultivated peanut BMC Plant Biol 2012 12 80 10.1186/1471-2229-12-80 22672714 PMC3404960

[B15] Singh AK Simpson CE Smart J Biosystematics and genetic resources, Chap.4 in the Groundnut crop: A scientific basis for improvement 1994 Chapman &Hall, London

[B16] Kochert G Stalker HT Gimenes M Galgaro L Lopes CR Moore K RFLP and cytogenetic evidence on the origin and evolution of allotetraploid domesticated peanut, Arachis hypogaea (Leguminosae) Am J Bot 1996 83 1282 1291 10.2307/2446112

[B17] Guyomarc'h H Sourdille P Charmet G Edwards KJ Bernard M Characterisation of polymorphic microsatellite markers from Aegilops tauschii and transferability to the D-genome of bread wheat Theor Appl Genet 2002 104 1164 1172 10.1007/s00122-001-0827-7 12582627

[B18] Gill KS Lubbers EL Gill BS Raupp WJ Cox TS A genetic linkage map of Triticum tauschii (DD) and its relationship to the D-genome of bread wheat (AABBDD) Genome 1991 34 362 374 10.1139/g91-058

[B19] Echt CS Kidwell KK Knapp SJ Osborn TC McCoy TJ Linkage mapping in diploid alfalfa (Medicago sativa) Genome 1993 37 61 71 7910147 10.1139/g94-008

[B20] Brummer EC Bouton JH Kochert G Development of an RFLP map in diploid alfalfa Theor Appl Genet 1993 86 329 332 10.1007/BF00222097 24193478

[B21] Yu GX Wise RP An anchored AFLP- and retrotransposon-based map of diploid Avena Genome 2000 43 736 749 11081962

[B22] Stalker HT Ferguson ME Valls JFM Pittman RN Simpson CE Bramel-Cox P Catalog of Arachis germplasm collection 2002 http://wwwicrisatorg/text/research/grep/homepage/groundnut/arachis/starthtm

[B23] USDA-ARS Germplasm Resources Information Network Species Records of Arachis 2011 http://wwwars-gringov/cgi-bin/npgs/html/splistpl?889

[B24] Stalker HT Kochert GD Dhesi JS Genetic diversity within the species Arachis duranensis Krapov. & W.C. Gregory, a possible progenitor of cultivated peanut Genome 1995 38 1201 1212 10.1139/g95-158 18470240

[B25] Robledo G Seijo G Species relationships among the wild B genome of Arachis species (section Arachis) based on FISH mapping of rDNA loci and heterochromatin detection: A new proposal for genome arrangement Theor Appl Genet 2010 121 1033 1046 10.1007/s00122-010-1369-7 20552326

[B26] Pandey MK Monyo E Ozias-Akins P Liang X Guimarães P Nigam SN Upadhyaya HD Janila P Zhang X Guo B Advances in Arachis genomics for peanut improvement Biotech Adv 2012 30 639 651 10.1016/j.biotechadv.2011.11.001 22094114

[B27] Gimenes MA Hoshino AA Barbosa AVG Palmieri DA Lopes CR Characterization and transferability of microsatellite markers of the cultivated peanut (Arachis hypogaea) BMC Plant Biol 2007 7 9 10.1186/1471-2229-7-9 17326826 PMC1829157

[B28] Mace ES Varshney RK Mahalakshmi V Seetha K Gafoor A Leeladevi Y Crouch JH In silico development of simple sequence repeat markers within the aeschynomenoid/dalbergoid and genistoid clades of the Leguminosae family and their transferability to Arachis hypogaea, groundnut Plant Sci 2008 174 51 60 10.1016/j.plantsci.2007.09.014

[B29] Proite K Leal-Bertioli S Bertioli D Moretzsohn M da Silva F Martins N Guimaraes P ESTs from a wild Arachis species for gene discovery and marker development BMC Plant Biol 2007 7 7 10.1186/1471-2229-7-7 17302987 PMC1808460

[B30] Cuc LM Mace ES Crouch JH Quang VD Long TD Varshney RK Isolation and characterization of novel microsatellite markers and their application for diversity assessment in cultivated groundnut (Arachis hypogaea) BMC Plant Biol 2008 8 55 10.1186/1471-2229-8-55 18482440 PMC2416452

[B31] Yuan M Gong LM Meng RH Li SL Dang P Guo BZ He GH Development of trinucleotide (GGC)n SSR markers in peanut (Arachis hypogaea L.) Electronic J Biotech 2010 13 6 6

[B32] Song GQ Li MJ Xiao H Wang XJ Tang RH Xia H Zhao CZ Bi YP EST sequencing and SSR marker development from cultivated peanut (Arachis hypogaea L.) Electronic J Biotech 2010 13 3 10

[B33] Wang CT Yang XD Chen DX Yu SL Liu GZ Tang YY Xu JZ Isolation of simple sequence repeats from groundnut Electronic J Biotech 2007 10 3 10

[B34] Liang XQ Chen XP Hong YB Liu HY Zhou GY Li SX Guo BZ Utility of EST-derived SSR in cultivated peanut (Arachis hypogaea L.) and Arachis wild species BMC Plant Biol 2009 9 35 10.1186/1471-2229-9-35 19309524 PMC2678122

[B35] Koilkonda P Sato S Tabata S Shirasawa K Hirakawa H Sakai H Sasamoto S Watanabe A Wada T Kishida Y Large-scale development of expressed sequence tag-derived simple sequence repeat markers and diversity analysis in Mol Breed 2012 30 125 138 10.1007/s11032-011-9604-8 22707912 PMC3362703

[B36] Bertioli DJ Moretzsohn MC Madsen LH Sandal N Leal-Bertioli SCM Guimaraes PM Hougaard BK Fredslund J Schauser L Nielsen AM An analysis of synteny of Arachis with Lotus and Medicago sheds new light on the structure, stability and evolution of legume genomes BMC Genomics 2009 10 45 10.1186/1471-2164-10-45 19166586 PMC2656529

[B37] Stalker HT Dhesi JS Parry D An analysis of the B genome species Arachis batizocoi (Fabaceae) Plant Syst Evol 1991 174 159 169 10.1007/BF00940337

[B38] Wells DE Gutierrez L Xu Z Krylov V Macha J Blankenburg KP Hitchens M Bellot LJ Spivey M Stemple DL A genetic map of Xenopus tropicalis Devel Biol 2011 354 1 8 10.1016/j.ydbio.2011.03.022 21458440 PMC3098391

[B39] Wu Y Bhat PR Close TJ Lonardi S Efficient and accurate construction of genetic linkage maps from the minimum spanning tree of a graph PLoS Genet 2008 4 10 10.1371/journal.pgen.0040010 PMC2556103 18846212

[B40] Heslop-Harrison JS Comparative genome organization in plants: From sequence and markers to chromatin and chromosomes Plant Cell 2000 12 617 635 10810139 10.1105/tpc.12.5.617 PMC139916

[B41] Paape T Zhou P Branca A Briskine R Young N Tiffin P Fine-scale population recombination rates, hotspots, and correlates of recombination in the Medicago truncatula genome Genome Biol Evol 2012 4 726 732 10.1093/gbe/evs046 22554552 PMC3381680

[B42] Leal-Bertioli SCM Jose ACVF ves-Freitas DMT Moretzsohn MC Guimaraes PM Nielen S Vidigal BS Pereira RW Pike J Favero AP Identification of candidate genome regions controlling disease resistance in Arachis BMC Plant Biol 2009 9 112 10.1186/1471-2229-9-112 19698131 PMC2739205

[B43] Nagy ED Chu Y Guo YF Khanal S Tang SX Li Y Dong WBB Timper P Taylor C Ozias-Akins P Recombination is suppressed in an alien introgression in peanut harboring Rma, a dominant root-knot nematode resistance gene Mol Breed 2010 26 357 370 10.1007/s11032-010-9430-4

[B44] Bowers JE Arias MA Asher R Avise JA Ball RT Brewer GA Buss RW Chen AH Edwards TM Estill JC Comparative physical mapping links conservation of microsynteny to chromosome structure and recombination in grasses Proc Natl Acad Sci USA 2005 102 13206 13211 10.1073/pnas.0502365102 16141333 PMC1201573

[B45] Murray MG Thompson WR Rapid isolation of high molecular weight plant DNA Nucl Acids Res 1980 8 4321 4325 10.1093/nar/8.19.4321 7433111 PMC324241

[B46] Doyle JJ Doyle JL A rapid DNA isolation procedure for small quantities of fresh leaf tissue Phytochem Bull 1987 19 11 15

[B47] Hopkins MS Casa AM Wang T Mitchell SE Dean RE Kochert GD Kresovich S Discovery and characterization of polymorphic simple sequence repeats (SSRs) in peanut Crop Sci 1999 39 1243 1247 10.2135/cropsci1999.0011183X003900040047x

[B48] Palmieri DA Bechara MD Curi RA Gimenes MA Lopes CR Novel polymorphic microsatellite markers in section Caulorrhizae (Arachis, Fabaceae) Mol Ecol Notes 2005 5 77 79 10.1111/j.1471-8286.2004.00838.x

[B49] Palmieri DA Hoshino AA Bravo JP Lopes CR Gimenes MA Isolation and characterization of microsatellite loci from the forage species Arachis pintoi (Genus Arachis) Mol Ecol Notes 2002 2 551 553 10.1046/j.1471-8286.2002.00317.x

[B50] He G Meng R Newman M Gao G Pittman RN Prakash CS Microsatellites as DNA markers in cultivated peanut (Arachis hypogaea L.) BMC Plant Biol 2003 3 3 10.1186/1471-2229-3-3 12713672 PMC155651

[B51] Ferguson ME Burow MD Schulze SR Bramel PJ Paterson AH Kresovich S Mitchell S Microsatellite identification and characterization in peanut (A. hypogaea L.) Theor Appl Genet 2004 108 1064 1070 10.1007/s00122-003-1535-2 15067392

[B52] Krishna GK Zhang J Burow M Pittman RN Delikostadinov SG Lu Y Puppala N Genetic diversity analysis in Valencia peanut (Arachis hypogaea L.) using microsatellite markers Cell Mol Biol Lett 2004 9 685 697 15647791

[B53] Moretzsohn MC Hopkins MS Mitchell SE Kresovich S Valls JF Ferreira ME Genetic diversity of peanut (Arachis hypogaea L.) and its wild relatives based on the analysis of hypervariable regions of the genome BMC Plant Biol 2004 4 11 10.1186/1471-2229-4-11 15253775 PMC491793

[B54] Minch E Ruiz-Linares A Goldstein D Feldman M Cavalli-Sforza LL MICROSAT: A computer program for calculating various statistics on microsatellite allele data, ver. 1.5d 1997 http://hpglstanfordedu/projects/microsat/

[B55] Felsenstein J PHYLIP - Phylogeny Inference Package (version 3.2) Cladistics 1989 5 164 166

[B56] Bertioli DJ Leal-Bertioli SCM Lion MB Santos VL Pappas G Cannon SB Guimaraes PM A large scale analysis of resistance gene homologues in Arachis Mol Genet Genom 2003 270 34 45 10.1007/s00438-003-0893-4 12928866

[B57] Yuksel B Estill JC Schulze SR Paterson AH Organization and evolution of resistance gene analogs in peanut Mol Genet Genom 2005 274 248 263 10.1007/s00438-005-0022-7 16179993

[B58] Orita M Iwahana H Kanazawa H Hayashi K Sekiya T Detection of polymorphisms of human DNA by gel-electrophoresis as single-strand conformation polymorphisms Proc Natl Acad Sci USA 1989 86 2766 2770 10.1073/pnas.86.8.2766 2565038 PMC286999

[B59] Sanguinetti CJ Neto ED Simpson AJG Rapid silver staining and recovery of PCR products separated on polyacrylamide gels Biotechniques 1994 17 914 7840973

[B60] Radwan O Gandhi S Heesacker A Whitaker B Taylor C Plocik A Kesseli R Kozik A Michelmore RW Knapp SJ Genetic diversity and genomic distribution of homologs encoding NBS-LRR disease resistance proteins in sunflower Mol Genet Genom 2008 280 111 125 10.1007/s00438-008-0346-1 18553106

[B61] Chu Y Holbrook CC Timper P Ozias-Akins P Development of a PCR-based molecular marker to select for nematode resistance in peanut Crop Sci 2007 47 841 847 10.2135/cropsci2006.07.0474

[B62] Chevreux B Pfisterer T Drescher B Driesel AJ Muller WEG Wetter T Suhai S Using the miraEST assembler for reliable and automated mRNA transcript assembly and SNP detection in sequenced ESTs Genome Res 2004 14 1147 1159 10.1101/gr.1917404 15140833 PMC419793

[B63] Van Ooijen JW Voorrips RE JoinMap 3.0 software for the calculation of genetic linkage maps 2001 Plant Research Internation, Wageningen, the Netherlands

[B64] Voorrips RE MapChart: software for the graphical presentation of linkage maps and QTLs J Hered 2002 93 77 78 10.1093/jhered/93.1.77 12011185

