# Supplementary figures and images for: A high-density genetic map of Arachis duranensis, a diploid ancestor of cultivated peanut
Source: BMC Genomics. 2012 Sep 11;13:469. doi: 10.1186/1471-2164-13-469 (PMC3542255; doi:10.1186/1471-2164-13-469)

6A

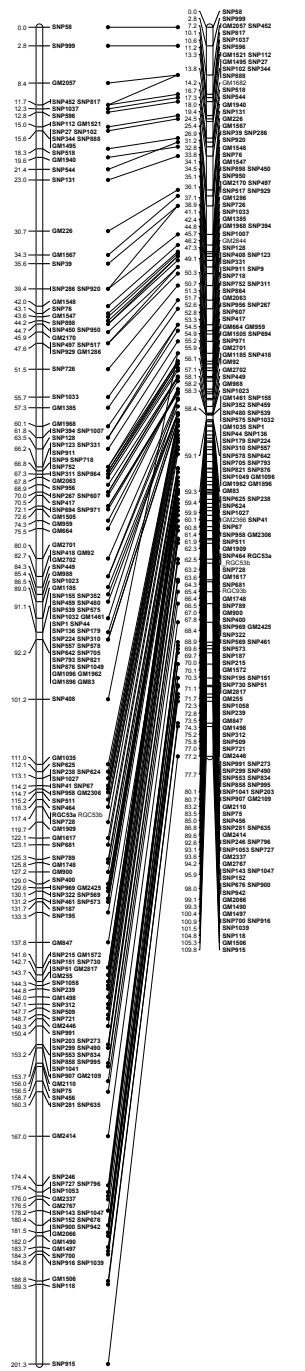

7A

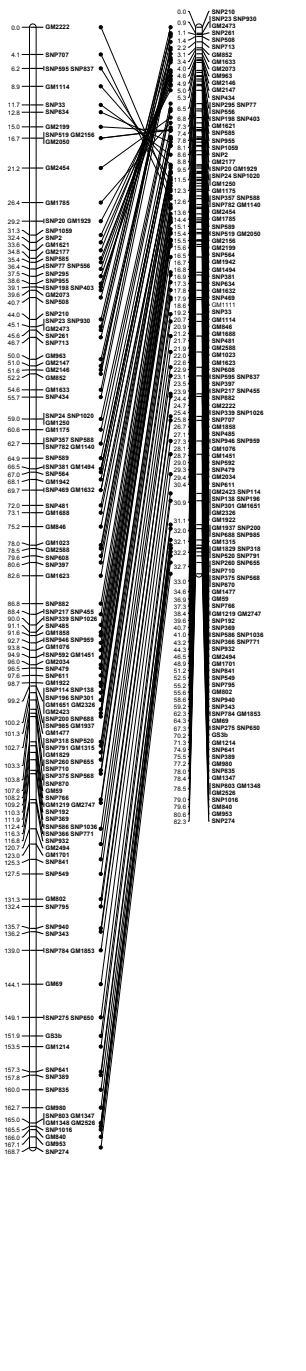

8A

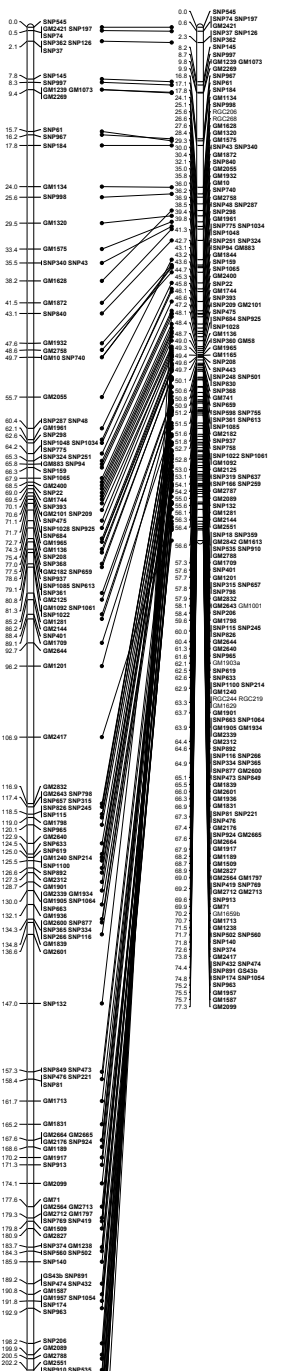

9A

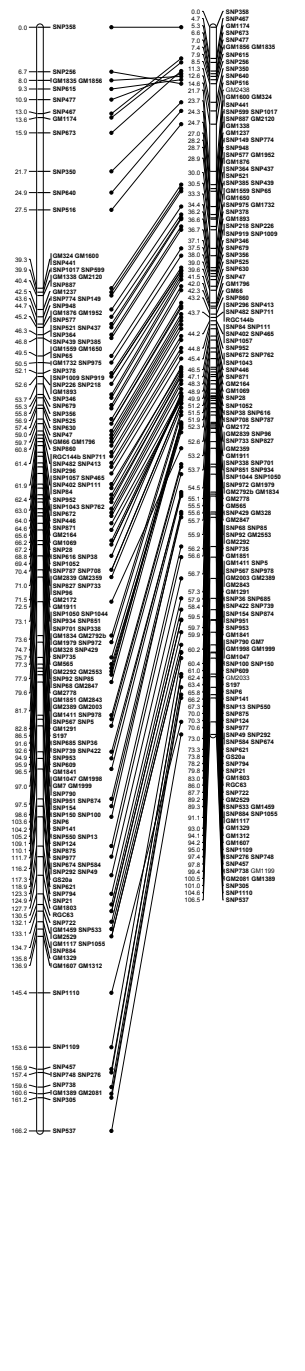

10A

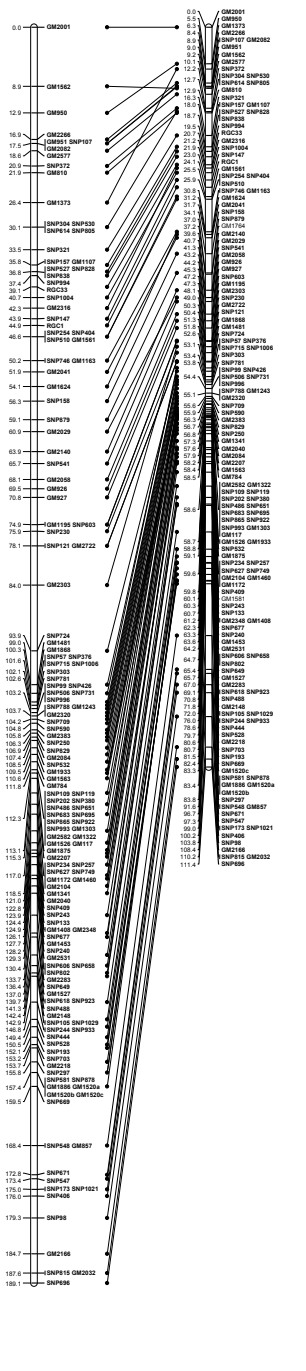

Supplement: Additional file 2 — Comparative genetic mapping ofArachis duranensisusing two different software programs on the same dataset. Genetic maps were constructed by MSTMap (left) using 1673 co-dominant markers and Joinmap 3.0 (right) using 1724 markers. Linkage group assignments, marker orders and genetic distances were highly consistent, except for the order among a few closely linked loci. Marker positions determined by Joinmap 3.0 are provided in Additional file 6. [file 1471-2164-13-469-S2.pdf]
